# Supplementary material for: A highly resolved integrated single-cell atlas of HPV-negative head and neck cancer
Source: bioRxiv. 2025 Mar 4:2025.03.02.640812. Preprint. [Version 1] doi: 10.1101/2025.03.02.640812 (PMC11908118; doi:10.1101/2025.03.02.640812)
Supplement: Supplement 1 [file NIHPP2025.03.02.640812v1-supplement-1.pdf]

bioRxiv preprint doi: https://doi.org/10.1101/2025.03.02.640812; this version posted March 4, 2025. The copyright holder for this preprint (which was not certified by peer review) is the author/funder, who has granted bioRxiv a license to display the preprint in perpetuity. It is made available under aCC-BY 4.0 International license.

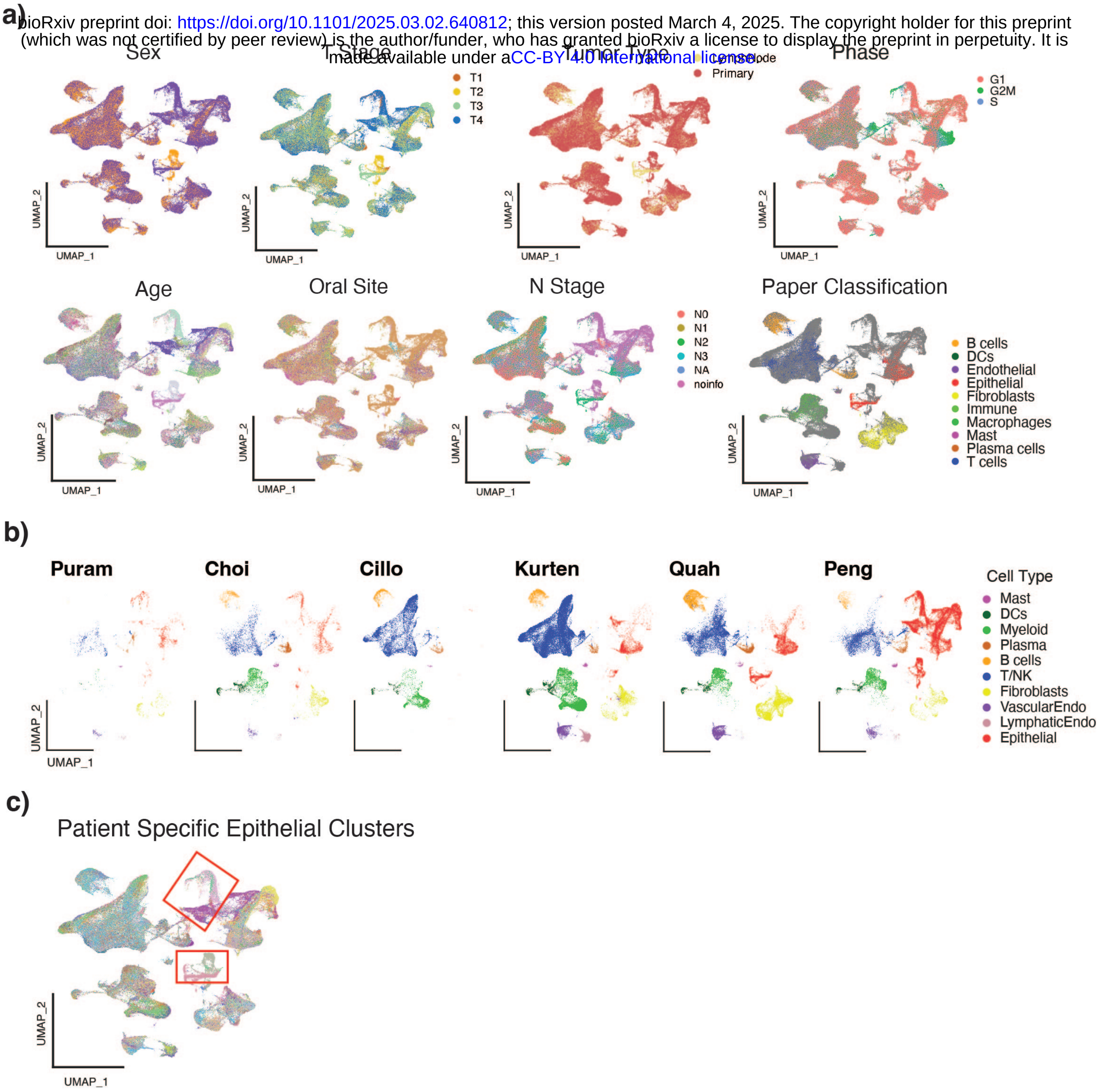

bioRxiv preprint doi: https://doi.org/10.1101/2025.03.02.640812; this version posted March 4, 2025. The copyright holder for this preprint (which was not certified by peer review) is the author/funder, who has granted bioRxiv a license to display the preprint in perpetuity. It is made available under aCC-BY 4.0 International license.

Supplementary Figure 1: Summary of Atlas Metadata. a) Visualization of metadata on atlas UMAP. b) Integrated UMAP split by dataset and colored by cell type. c) Atlas UMAP colored by patient. Red boxes highlight epithelial or salivary clusters with most cells originating from one patient.

bioRxiv preprint doi: https://doi.org/10.1101/2025.03.02.640812; this version posted March 4, 2025. The copyright holder for this preprint (which was not certified by peer review) is the author/funder, who has granted bioRxiv a license to display the preprint in perpetuity. It is made available under aCC-BY 4.0 International license.

a)

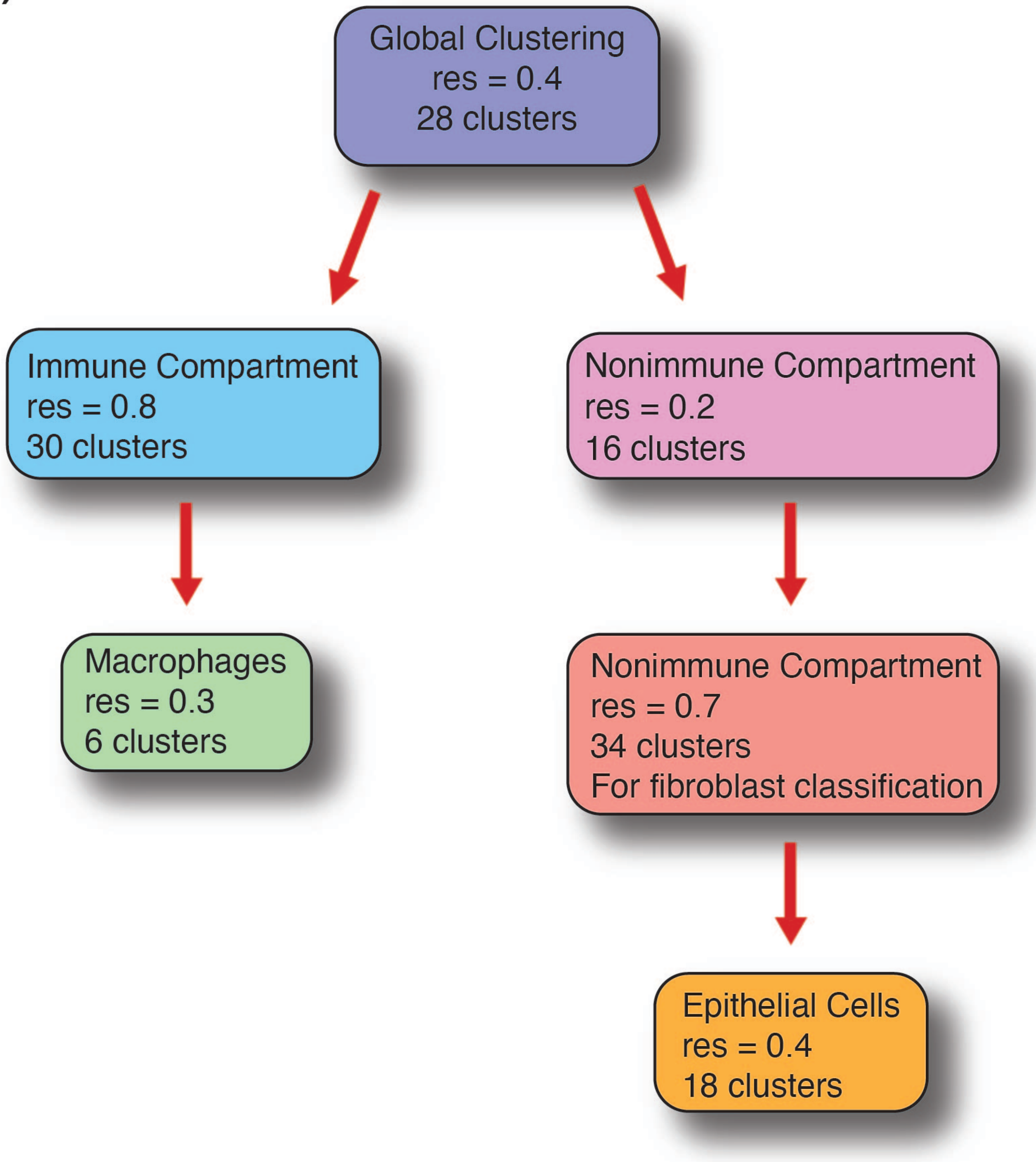

bioRxiv preprint doi: https://doi.org/10.1101/2025.03.02.640812; this version posted March 4, 2025. The copyright holder for this preprint (which was not certified by peer review) is the author/funder, who has granted bioRxiv a license to display the preprint in perpetuity. It is made available under aCC-BY 4.0 International license.

Supplementary Figure 2: Overview of clustering performed in different compartments at various resolutions and number of clusters found.

bioRxiv preprint doi: https://doi.org/10.1101/2025.03.02.640812; this version posted March 4, 2025. The copyright holder for this preprint (which was not certified by peer review) is the author/funder, who has granted bioRxiv a license to display the preprint in perpetuity. It is made available under aCC-BY 4.0 International license.

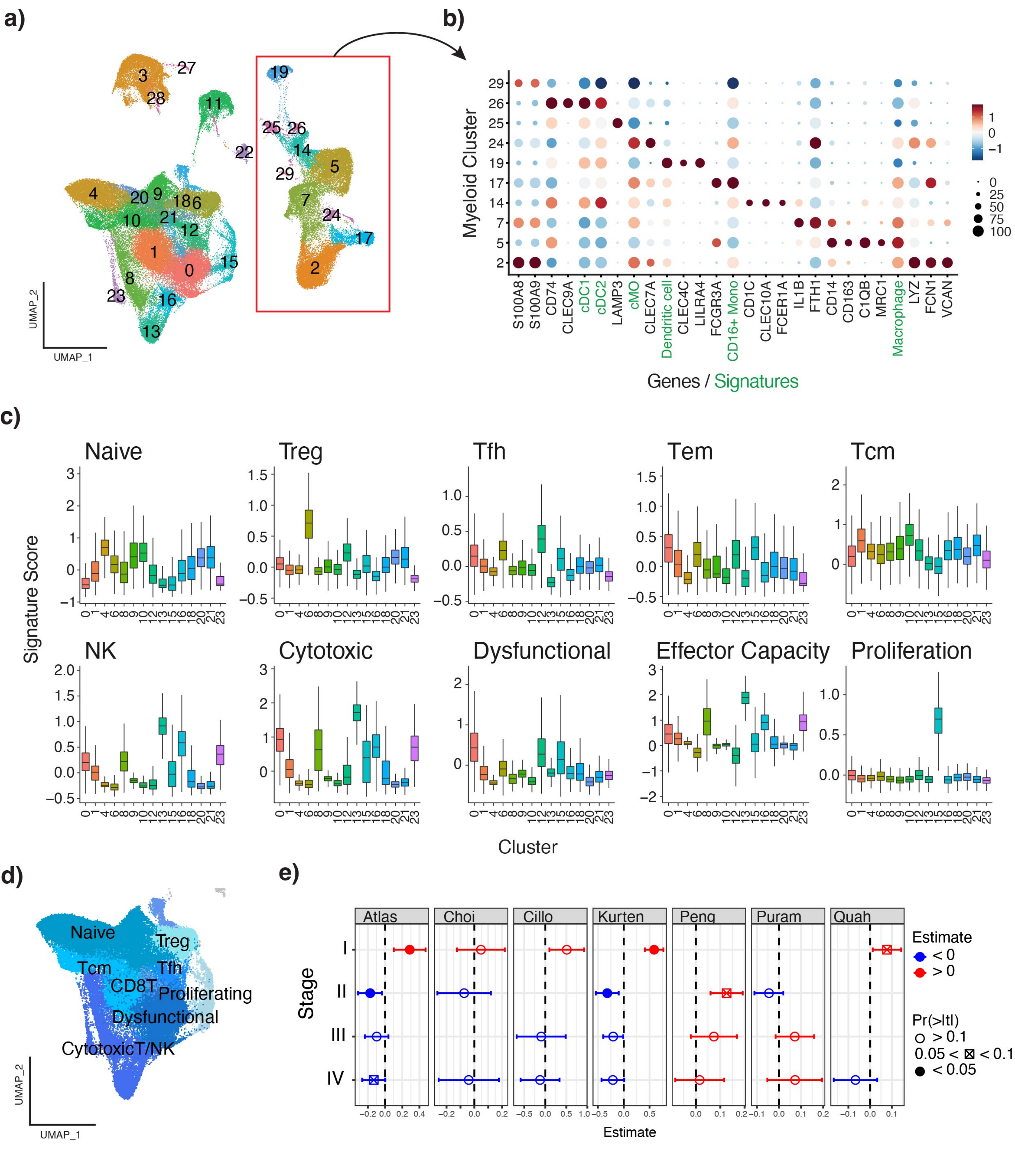

bioRxiv preprint doi: https://doi.org/10.1101/2025.03.02.640812; this version posted March 4, 2025. The copyright holder for this preprint (which was not certified by peer review) is the author/funder, who has granted bioRxiv a license to display the preprint in perpetuity. It is made available under aCC-BY 4.0 International license.

Supplementary Figure 3: Identification of T cell and myeloid cell subpopulations and association with stage. a) UMAP of the immune compartment clustered at resolution 0.8. b) Dotplot of marker genes (black) and signatures (green) enriched in the myeloid clusters. c) Boxplots showing the module score of different T and NK cell signatures across the T and NK cell clusters. d) Summary of T cell classification based on signature enrichment of the T cells. e) Estimates for the association of T cell effector capacity score with stage across all datasets in the atlas (left), followed by within each dataset separately.

bioRxiv preprint doi: https://doi.org/10.1101/2025.03.02.640812; this version posted March 4, 2025. The copyright holder for this preprint (which was not certified by peer review) is the author/funder, who has granted bioRxiv a license to display the preprint in perpetuity. It is made available under aCC-BY 4.0 International license.

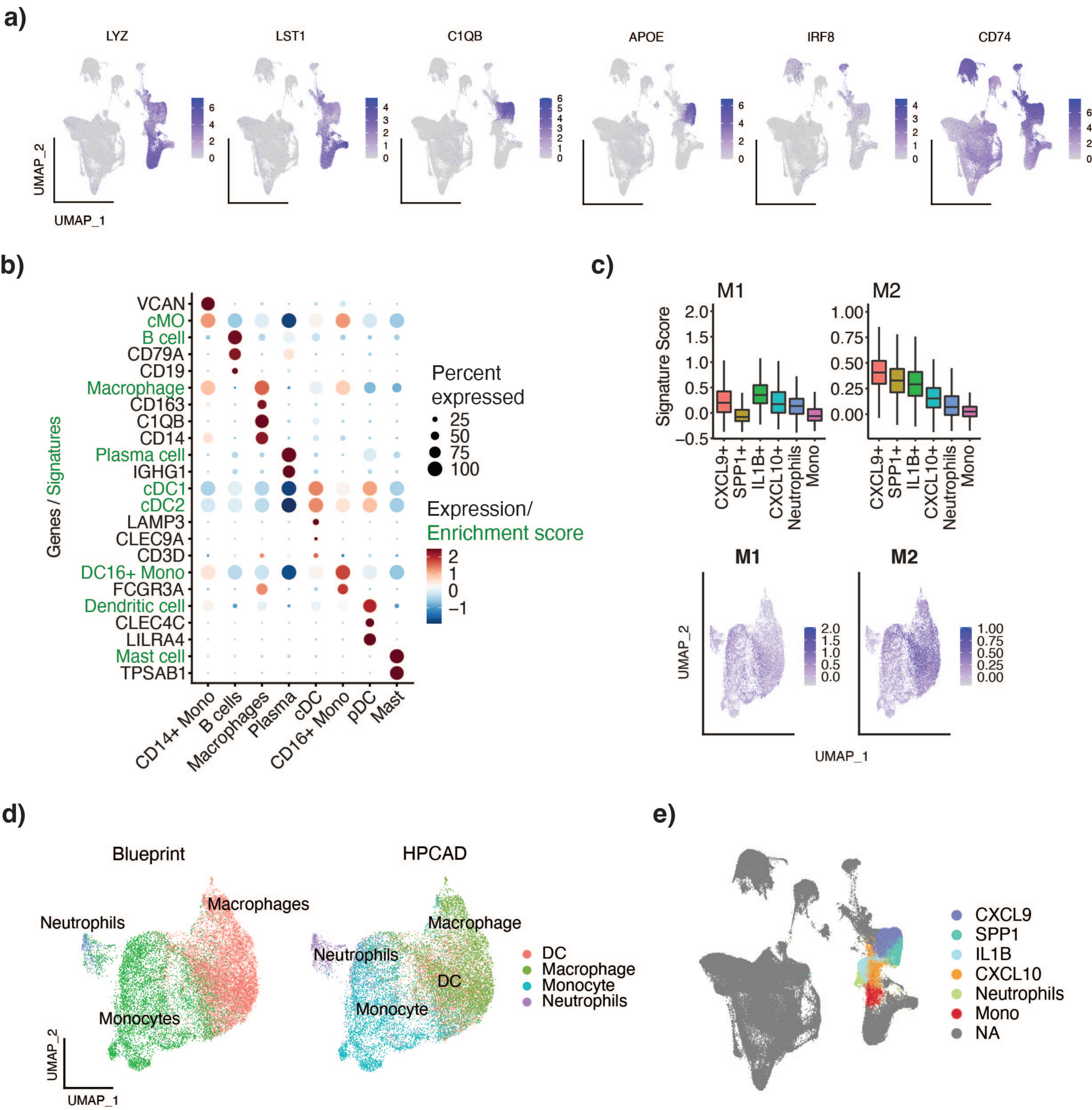

bioRxiv preprint doi: https://doi.org/10.1101/2025.03.02.640812; this version posted March 4, 2025. The copyright holder for this preprint (which was not certified by peer review) is the author/funder, who has granted bioRxiv a license to display the preprint in perpetuity. It is made available under aCC-BY 4.0 International license.

Supplementary Figure 4: Macrophage cell classification. a) UMAP of the immune compartment showing genes influential in myeloid K2 Taxonomer clustering. From left to right: LYZ, LST1, C1QB, APOE, IRF8, CD74. b) Signature (green) and gene (black) expression in myeloid clusters. c) M1 and M2 signature score in macrophage subclusters. d) Blueprint and HPCAD cell classifications of top cell types identified. Supplementary table 3 contains all classifications. e) Immune compartment UMAP colored by myeloid sub-clusters identified in Fig 3.

bioRxiv preprint doi: https://doi.org/10.1101/2025.03.02.640812; this version posted March 4, 2025. The copyright holder for this preprint (which was not certified by peer review) is the author/funder, who has granted bioRxiv a license to display the preprint in perpetuity. It is made available under aCC-BY 4.0 International license.

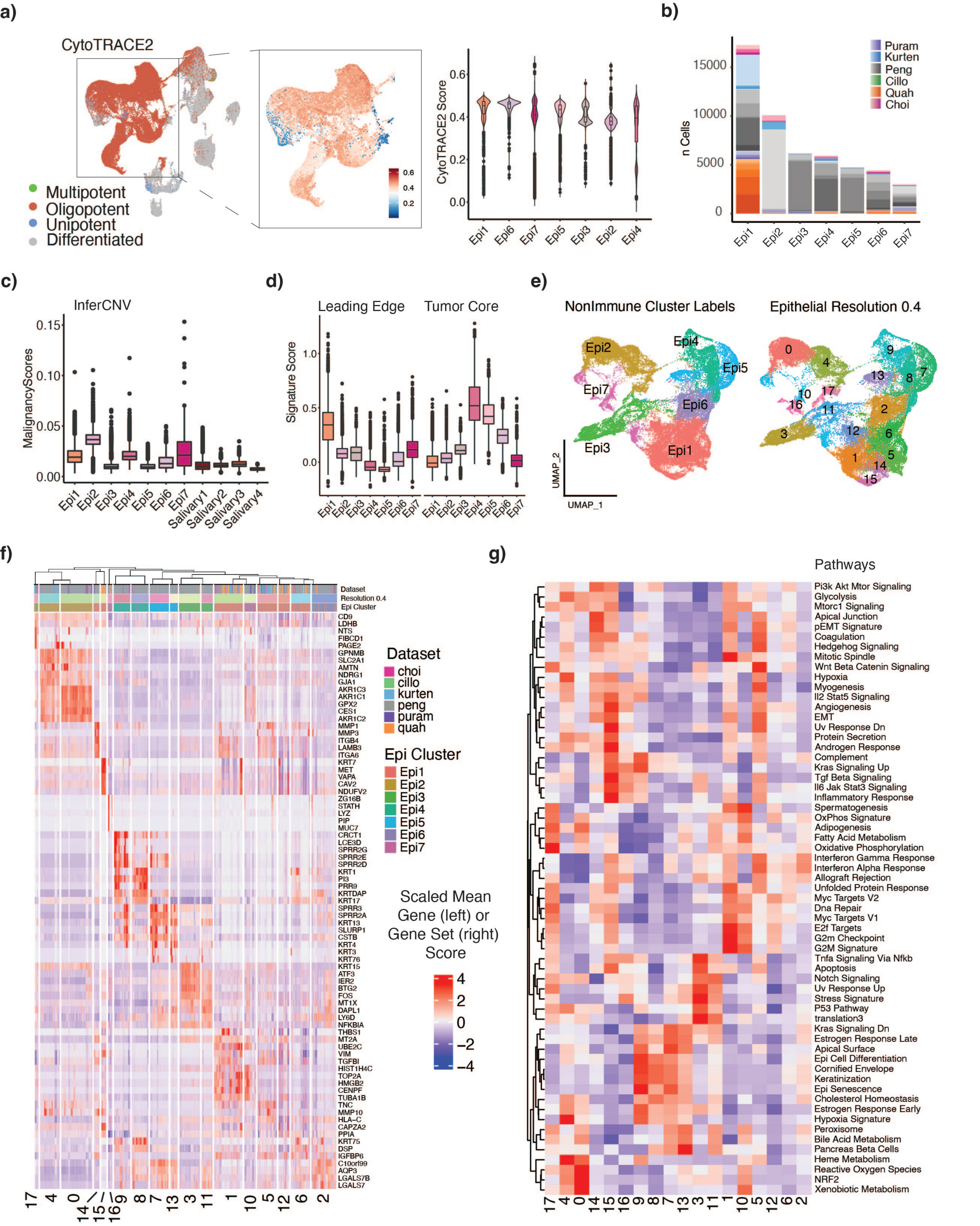

bioRxiv preprint doi: https://doi.org/10.1101/2025.03.02.640812; this version posted March 4, 2025. The copyright holder for this preprint (which was not certified by peer review) is the author/funder, who has granted bioRxiv a license to display the preprint in perpetuity. It is made available under aCC-BY 4.0 International license.

Supplementary Figure 5. High-resolution characterization of the epithelial cells. a) CytoTRACE2 potency classification (left) across epithelial clusters, and scores (UMAP middle and violin plots right) across epithelial clusters. b) Number of cells per patient per cluster. Each dataset is a color, which each patient a different shade within the color group. c) InferCNV malignancy scores across clusters. d) Boxplots showing enrichment scores of Arora et al.'s leading edge and tumor core signatures across epithelial clusters. e) Epithelial cells reclustered and labelled by initial whole-immune compartment clustering (left) and by higher resolution 0.4 clustering (right). f) Heatmap of top five genes per cluster at resolution 0.4. g) Heatmap of enrichment scores of Hallmark genesets and signatures from Puram et al. (2017) per each cluster found at resolution 0.4.

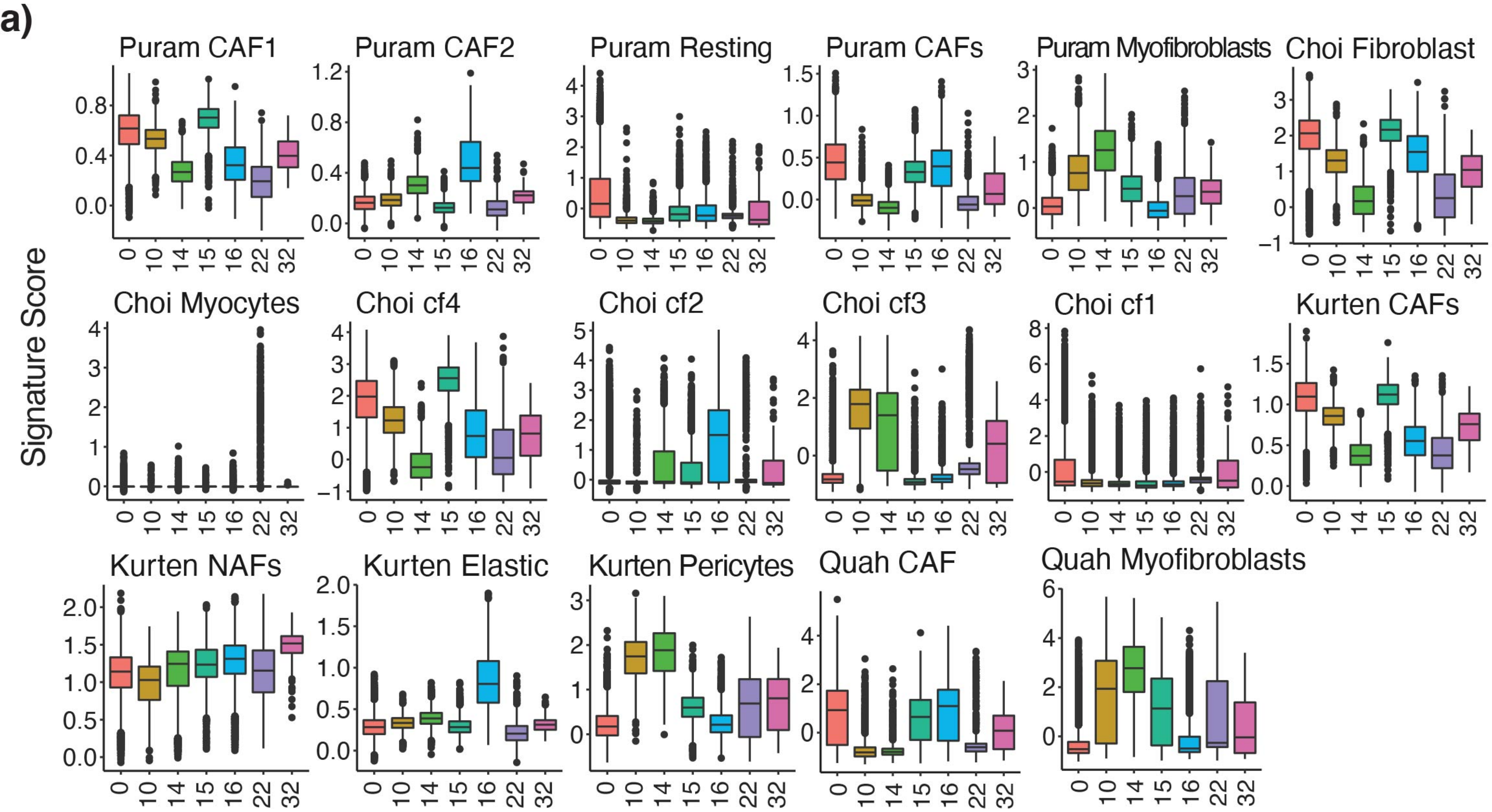
a)
Signature Score
Puram CAF1
Puram CAF2
Puram Resting
Puram CAFs
Puram Myofibroblasts
Choi Fibroblast
Choi Myocytes
Choi cf4
Choi cf2
Choi cf3
Choi cf1
Kurten CAFs
Kurten NAFs
Kurten Elastic
Kurten Pericytes
Quah CAF
Quah Myofibroblasts
0
10
14
15
16
22
32

bioRxiv preprint doi: https://doi.org/10.1101/2025.03.02.640812; this version posted March 4, 2025. The copyright holder for this preprint (which was not certified by peer review) is the author/funder, who has granted bioRxiv a license to display the preprint in perpetuity. It is made available under aCC-BY 4.0 International license.

Supplementary Figure 6. Reconciliation of fibroblast terminology. a) Enrichment of fibroblast signatures from original publications in seven fibroblast clusters.

bioRxiv preprint doi: https://doi.org/10.1101/2025.03.02.640812; this version posted March 4, 2025. The copyright holder for this preprint (which was not certified by peer review) is the author/funder, who has granted bioRxiv a license to display the preprint in perpetuity. It is made available under aCC-BY 4.0 International license.

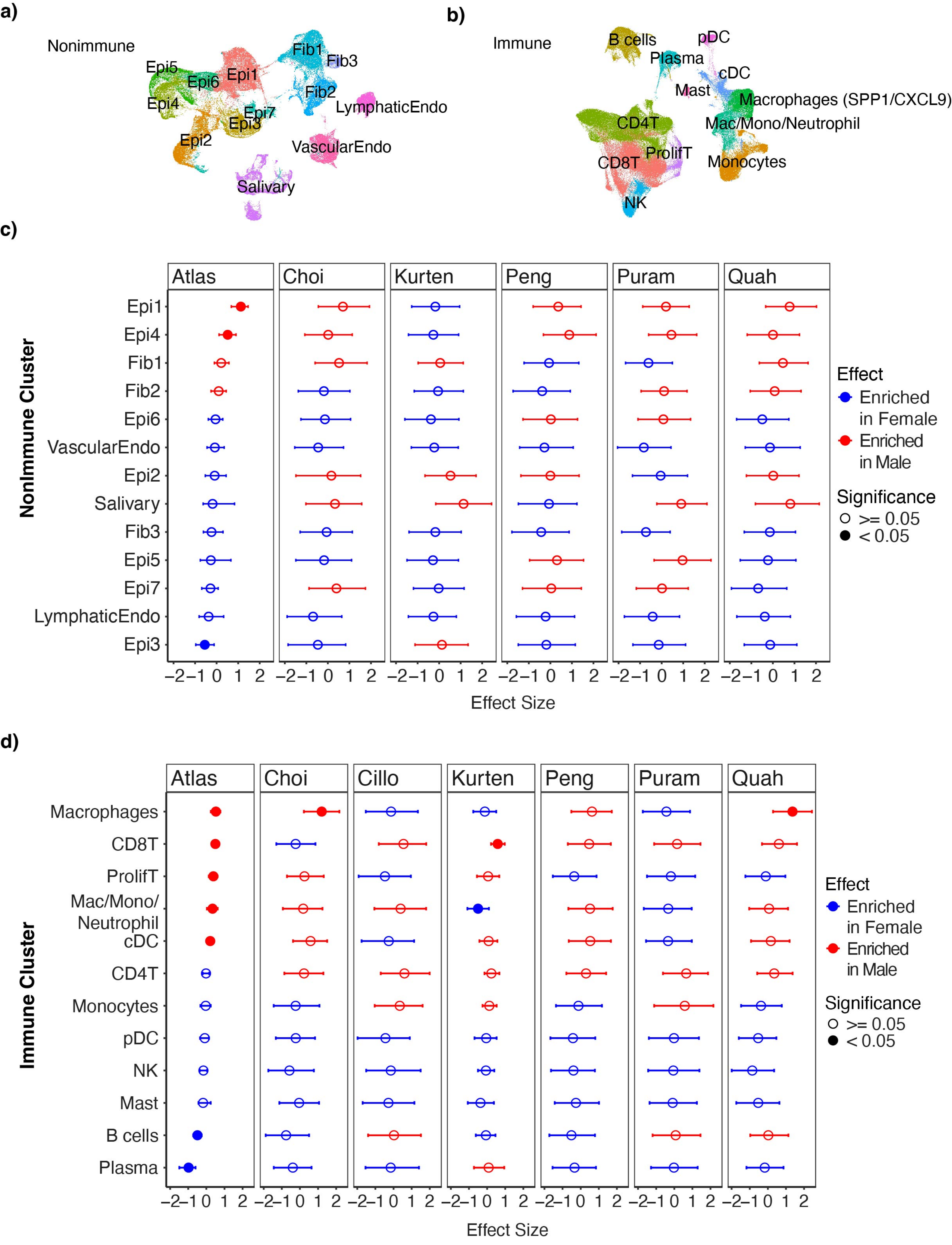

bioRxiv preprint doi: https://doi.org/10.1101/2025.03.02.640812; this version posted March 4, 2025. The copyright holder for this preprint (which was not certified by peer review) is the author/funder, who has granted bioRxiv a license to display the preprint in perpetuity. It is made available under aCC-BY 4.0 International license.

Supplementary Figure 7. Sex-specific cell type proportion changes on a dataset level. Clustering in the a) nonimmune and b) immune compartments used as input to sccomp. c) Cell type proportion changes identified by sccomp within the nonimmune compartment when using the whole atlas (left), and individually within each dataset. d) Cell type proportion changes identified by sccomp within the immune compartment when using the whole atlas (left), and individually within each dataset.
